# Name-calling in the hippocampus (and beyond): coming to terms with neuron types and properties

**DOI:** 10.1007/s40708-016-0053-3

**Published:** 2016-06-09

**Authors:** D. J. Hamilton, D. W. Wheeler, C. M. White, C. L. Rees, A. O. Komendantov, M. Bergamino, G. A. Ascoli

**Affiliations:** 1 Center for Neural Informatics, Structure, & Plasticity, Molecular Neuroscience Dept., Krasnow Institute for Advanced Study, MS2A1, George Mason University, Fairfax, VA 22030-4444 USA; 2 Laureate Institute for Brain Research, 6655 S Yale Ave, Tulsa, OK 74136 USA

**Keywords:** Hippocampus, Neuron, Type, Property, Nomenclature

## Abstract

Widely spread naming inconsistencies in neuroscience pose a vexing obstacle to effective communication within and across areas of expertise. This problem is particularly acute when identifying neuron types and their properties. Hippocampome.org is a web-accessible neuroinformatics resource that organizes existing data about essential properties of all known neuron types in the rodent hippocampal formation. Hippocampome.org links evidence supporting the assignment of a property to a type with direct pointers to quotes and figures. Mining this knowledge from peer-reviewed reports reveals the troubling extent of terminological ambiguity and undefined terms. Examples span simple cases of using multiple synonyms and acronyms for the same molecular biomarkers (or other property) to more complex cases of neuronal naming. New publications often use different terms without mapping them to previous terms. As a result, neurons of the same type are assigned disparate names, while neurons of different types are bestowed the same name. Furthermore, non-unique properties are frequently used as names, and several neuron types are not named at all. In order to alleviate this nomenclature confusion regarding hippocampal neuron types and properties, we introduce a new functionality of Hippocampome.org: a fully searchable, curated catalog of human and machine-readable definitions, each linked to the corresponding neuron and property terms. Furthermore, we extend our robust approach to providing each neuron type with an informative name and unique identifier by mapping all encountered synonyms and homonyms.

## Introduction

From its beginning, neuroscience has been tied to ad hoc neuron naming, which is subject to the whims of researchers with diverse interests. It has always been the inclination of neuroscientists to name neurons based on certain observed properties. Already in the 1800s, researchers leveraged ongoing progress in optical microscopy and newly discovered staining techniques to identify neuron types and their morphological features. Historical examples include Betz’ naming of “giant pyramids” [1] and Cajal’s description of “psychic cells” (nowadays known as pyramidal neurons) as characterized by “…a dendritic shaft and tuft directed toward the cerebral surface [and] the existence of collateral spines on the dendritic processes…” [2]. Thousands of reports describing neurons and their characteristics have been published since, and several dozens of distinct types of neurons had been already recognized before the turn of the millennium in each of several prominent neural systems, such as among the “GABAergic non-principal cells” of the hippocampus [3].

The often subjective and arbitrary naming of neurons led to a cluttered literature landscape in which breakdowns in communication can hinder the understanding of the structure and function of the brain. A comprehensive solution would require establishing a broadly applicable and widely accepted classification scheme defining neuron types based on their properties. However, despite early efforts focused on identifying key neuronal properties with precise terminology [4], to this date there is a high level of disorganization when it comes to reporting neuronal property information. Although community efforts exist for the expert curation of neuroanatomical terms pertaining to brain regions [5] and grass root scholarly collation of neuroscience terminology [6], the continuously increasing pace of data acquisition is paradoxically yielding an ever more fractured lexicon, creating serious impediment to progress.

We have previously proposed an ontological approach to defining neurons based on necessary and sufficient part-relation-value triple-store techniques [7]. In the absence of comprehensive data and unbiased sampling, however, it may be impossible to select a priori the appropriate defining properties [8]. Using too few or too many constraints results in under-defining or over-defining a neuron type. The former case (“over-lumping”) leads to a few large groups of neurons that share very few properties; the latter (“over-splitting”) leads to myriad types of doubtful interpretation. To complicate this matter further, the continuous gradation of key properties may require a shift to fuzzy classification approaches [9].

A recent empirical assessment of inter-investigator agreement on morphological classes of neocortical interneurons demonstrated a variable level of consensus across neuron types and properties [10]. One of the most reliable identifiers of neuron types is the presence or absence of axons and dendrites within well-defined neuroanatomical boundaries. Spotlighting this, Hippocampome.org [11] recently established unambiguous definitions of neuron types primarily based on axonal and dendritic distributions across all the main subregions and layers of the hippocampal formation. This classification approach yielded an initial catalog of 122 neuron types identified from the scientific literature. It is important to stress that the classification criteria employed by Hippocampome.org operate independently of previously used names.

In this framework, a neuron type is initially identified by its (putative) neurotransmitter and the presence of axons and dendrites in the distinct layers of dentate gyrus, CA3, CA2, CA1, subiculum, and entorhinal cortex. Each type is further characterized by available information on biomarker expression and electrophysiological features. This relatively simple characterization allows dense curation of the published literature through text mining and annotation. The resulting information is instantiated as a machine-readable electronic relational knowledge base that is publicly and freely available, facilitating web accessibility and computational analytics. With critical properties compiled in an easily accessible portal, Hippocampome.org provides a unique opportunity to establish a consistent set of definitions and a naming protocol that could be expanded to other cortical areas, aiding research and scientific communication.

The remaining of this report is organized as the following. The next section provides illustrative examples of the terminological confusion regarding neuron types and properties from the hippocampal literature. The following section outlines the three steps toward a solution: first, we describe the design of a database to define, store, browse, search, and retrieve human-interpretable but machine-readable definitions of neuron types based on their properties, as recently implemented at Hippocampome.org. Second, we introduce a newly deployed functionality that maps all relevant property terms to corresponding concepts, linking their occurrence in the published evidence to community-accepted definitions. Third, we offer a formal definition of the resulting neuron types and detail the process to assign each of them with a unique common name. The last section closes the paper with concluding remarks.

## A neuronal “Tower of Babel”

The nomenclatures of neuron types and of their features are both vexed with ambiguities, resulting in a “many-to-many” mapping between neurons and names as well as inconsistent definitions of properties. We illustrate below representative examples of the most common scenarios from the hippocampal literature.

When neurons are described in a publication, they are typically named in isolation, out of context with respect to the rest of the brain circuit and the literature. Sometimes neuron types or individual neurons are indicated solely by a non-descriptive label (e.g., “Type I” cells [12] or “cell #7” [13], and occasionally they are not named at all. When proper terms are used, it may still be difficult to discern whether a word is meant to be a name or merely a description, as when referring to “multipolar cells” [12, 14]. The result is often a baffling web of associations between names and neuron types.

Consider for instance the term “CA1 Bistratified cell originally chosen over 20 years ago to name a group of hippocampal neurons with axons and dendrites prominently invading the oriens and radiatum layers without crossing into lacunosum-moleculare [15]. Different authors later used the exact same noun referring to the morphological pattern of a different neuron type with axons distributed in the CA1 oriens and radiatum layers (though also extending into the subiculum), but dendrites limited to oriens [16]. Unfortunately, neurons with these distinct characteristics had already been bestowed the different name of “CA1 trilaminar cells” in an earlier article [17]. Nevertheless, the label “CA1 trilaminar cell” was also used to describe yet another neuron type that had a similar axonal distribution, but dendrites invading lacunosum-moleculare [16]. But the confusion does not end here, as other labs independently referred to this latter morphology as either “CA1 Schaffer-associated” [18] or “CA1 apical dendrite innervating” [19].

**Fig. 1:**
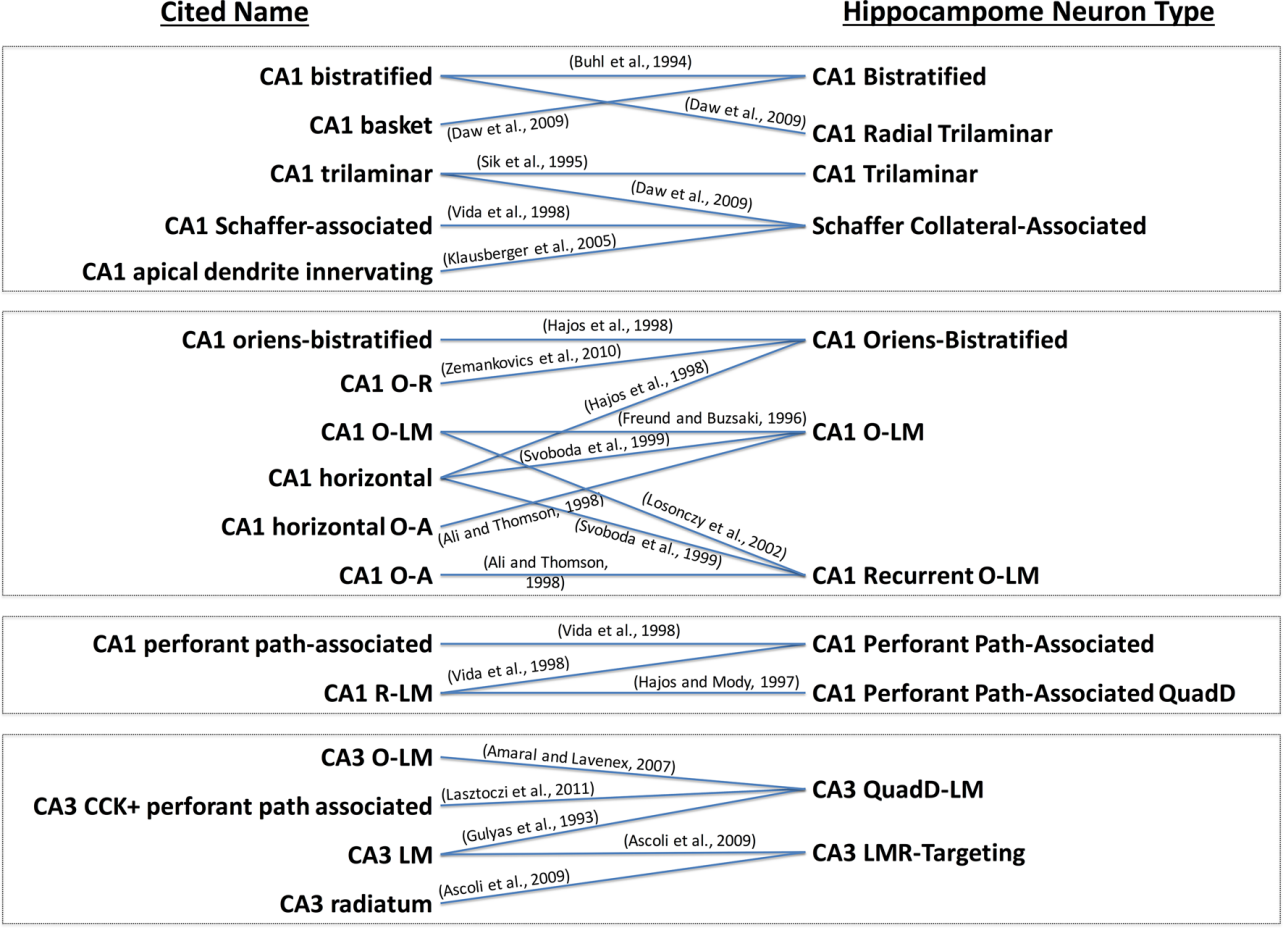
Relationships between cited names [3, 15–29] and neuron types. This* bipartite graph* highlights the naming confusion that is typical within the neuroscience community today

**Fig. 2:**
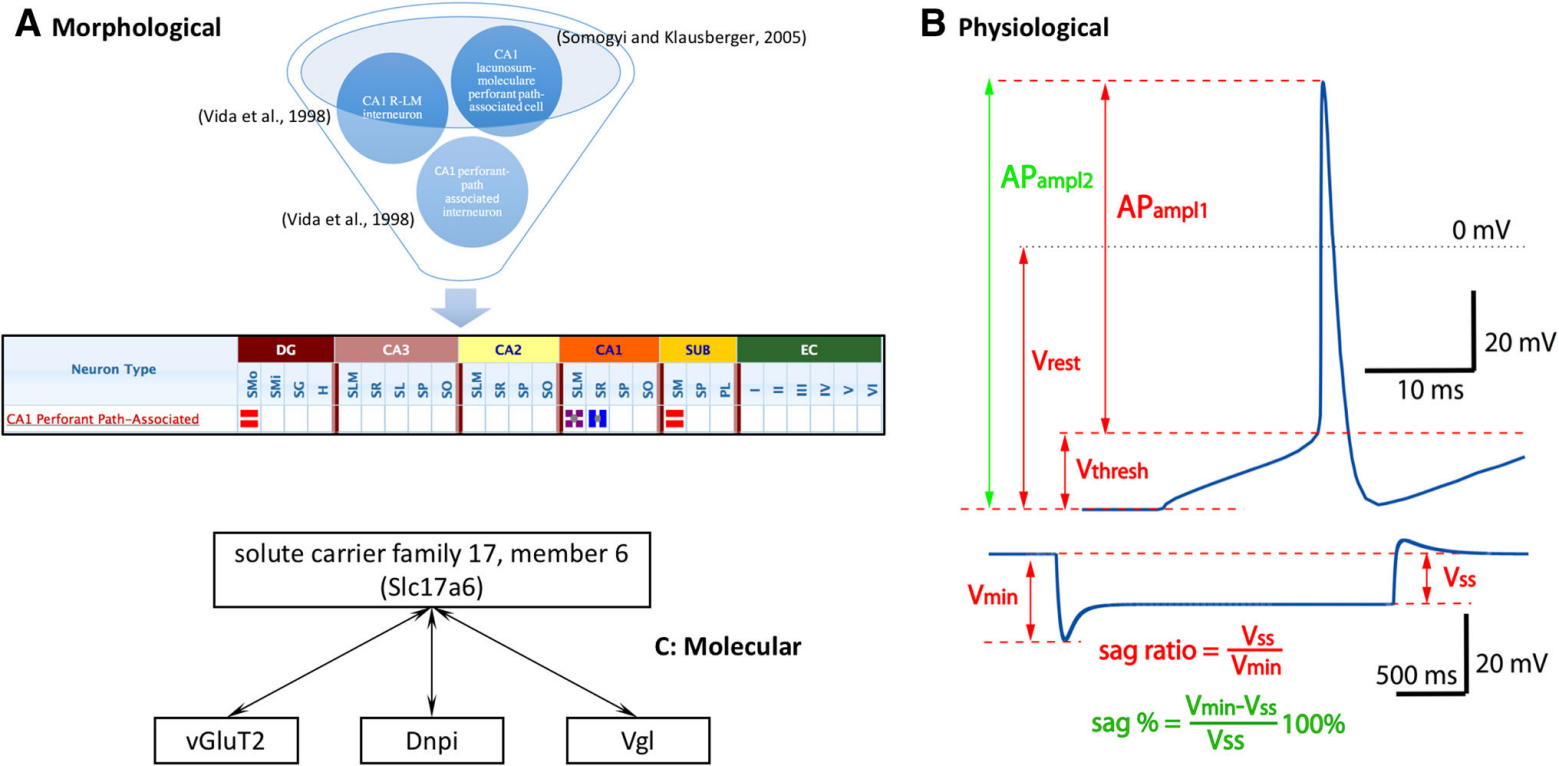
Examples of confusing nomenclature. **a** Morphological terms [18, 47]. **b** Physiological properties. Neuronal responses to suprathreshold depolarizing (*top*) and hyperpolarizing (*bottom*) current injections. *Green* and *red labels* show different definitions of electrophysiological parameters (action potential amplitude and sag ratio). *Vrest* resting membrane potential, *Vthresh* threshold potential, *Vmin* minimum of membrane potential drop, *Vss* steady‐state membrane potential under long‐lasting hyperpolarizing stimulation, *APampl* action potential amplitude. **c** Molecular terminology

We should note that these are not exceptional instances, but absolutely frequent occurrences, as depicted by several additional examples in Fig. 1 [20–29]. There are also multiple cases of the same referencing article calling a morphologically defined type by different synonyms, such as “perforant path-associated” and “CA1 R-LM” referring to neurons with axons and dendrites in CA1 stratum lacunosum-moleculare and dendrites in radiatum [18] (Fig. 2a). At the same time, these are not sterile spelling quibbles, because the specific laminar pattern of dendrites and axons defines the potential connectivity of the circuit and therefore the computational functions of neurons.

The confusion is not limited to neuron types but also affects the nomenclature of neuronal features, including morphological, electrophysiological, and molecular terminology. Qualitative phraseology is especially common in reporting morphological properties. An examination of the evidence collated in Hippocampome.org pertaining to the relative abundance of axons in an anatomical location of interest reveals ample use of terms such as “most,” “majority,” and “usually.” Furthermore, categorical terms are often employed to indicate continuous spatial distributions, as in “superficial/deep layer X,” “proximal/distal area Y,” and “septal/temporal region Z.” A clear consensus of how such terms should be adopted and interpreted, and what terms are to be avoided, reduces ambiguity. Hippocampome.org proposes a set of protocols for the description of neurites and their locations (hippocampome.org/full-interp).

The electrophysiological lexicon suffers not only from ambiguous descriptors but also from inconsistent definitions of the parameters themselves. For example, some investigators measure action potential amplitude from the resting membrane potential to the peak of the spike [30]. A complementary subset of studies, however, calculates action potential amplitude relative to the spike threshold potential [31]. The relationship between the minimum and the steady-state membrane potentials resulting from a hyperpolarizing current is similarly ambiguous. The sag ratio quantifies the relative difference between the peak hyperpolarization and steady-state hyperpolarization [32]. Alternatively, the sag percentage reports the fractional change in membrane potential from peak to steady state relative to the steady state [33]. Figure 2b schematically shows the differences between these parameter definitions. Plainly, the use of identical or similar names for terms with different electrophysiological meanings can lead to the propagation of confusion and, worse, incorrect interpretations of data that are incorporated into the literature moving forward.

Molecular biomarkers bear an overabundance of synonyms, homonyms, hyponyms, hypernyms, and abbreviations. There is movement toward standardizing the naming of proteins, but it is debatable whether the efforts are alleviating or augmenting confusion. For instance, the entire family of mammalian neuronal transporters has been given the official name of “solute carrier family [X] member [Y].” The new names confer that the proteins are transporters, but provide little information beyond that. As an example, some authors now refer to vesicular glutamate transporter 2 (Gene ID: 84487, ncbi.nlm.nih.gov/protein/NP_445879.1) by the abbreviation Slc17a6, short for the official full name “solute carrier family 17, member 6,” while others keep the familiar vGluT2. If these two alternatives were not enough, the marker is also known by the symbols Dnpi and Vgl [34–37] (Fig. 2c).

One of the worst cases of molecular biomarker terminology confusion in neuroscience involves glutamate receptors. Metabotropic glutamate receptors (mGluRs) are not to be confused with three classes of ionotropic receptors (GluRs): AMPA, kainate, and NMDA, sometimes referred to as AMPARs, KAs, and the NRs [38]. In the promising new naming schema for glutamate receptors, metabotropic receptors retain use of mGluR, while AMPA receptors use GluA, kainate GluK, and NMDA GluN [39]. It is yet to be seen how widely used either of these schemata will be. Alas, even if the entire research community compactly embraced them today, the problem of linking new information with previous publications would remain.

## Resolving the neuron-type crossword puzzle

The solution to both the naming dilemma and property-based neuronal classification lies in establishing and consistently applying an unambiguous, clearly defined, unique nomenclature with links to antecedent synonyms. With property terms, scholarly resources can serve as broadly accepted references and dictionaries, such as the Medical Subject Headings (MeSH) by the US National Library of Medicine [40] and NeuroLex by the National Institutes of Health-contracted Neuroscience Information Framework [41]. However, using such services requires turning attention away from the material with the confusing or unknown term, navigating external web site(s), finding and processing the definition(s), then refocusing attention to the original material. A terms portal integrated into the original material would greatly simplify the process.

### Data schema for property-based classification of hippocampal neurons

To solve the neuronal naming problem, the neuroscience community would ideally adopt a robust approach to classification. Using the distributions of axons and dendrites across identifiable anatomical areas is advantageous for a number of reasons. Axonal and dendritic patterning is fundamental to all neurons, yet sufficiently information-rich to allow grouping at a useful level of abstraction on the spectrum from considering all neurons the same (as would be the case if spike integrator were the chosen property) and each individual neuron unique (as would result if using exact matches of the neurite arbors). In addition, neurite patterns are more stable and less dependent upon experimental conditions than molecular markers and electrophysiology, respectively. Lastly, as demonstrated below, this approach naturally provides the means of creating unique, concise, informative names of neuron types.

**Fig. 3:**
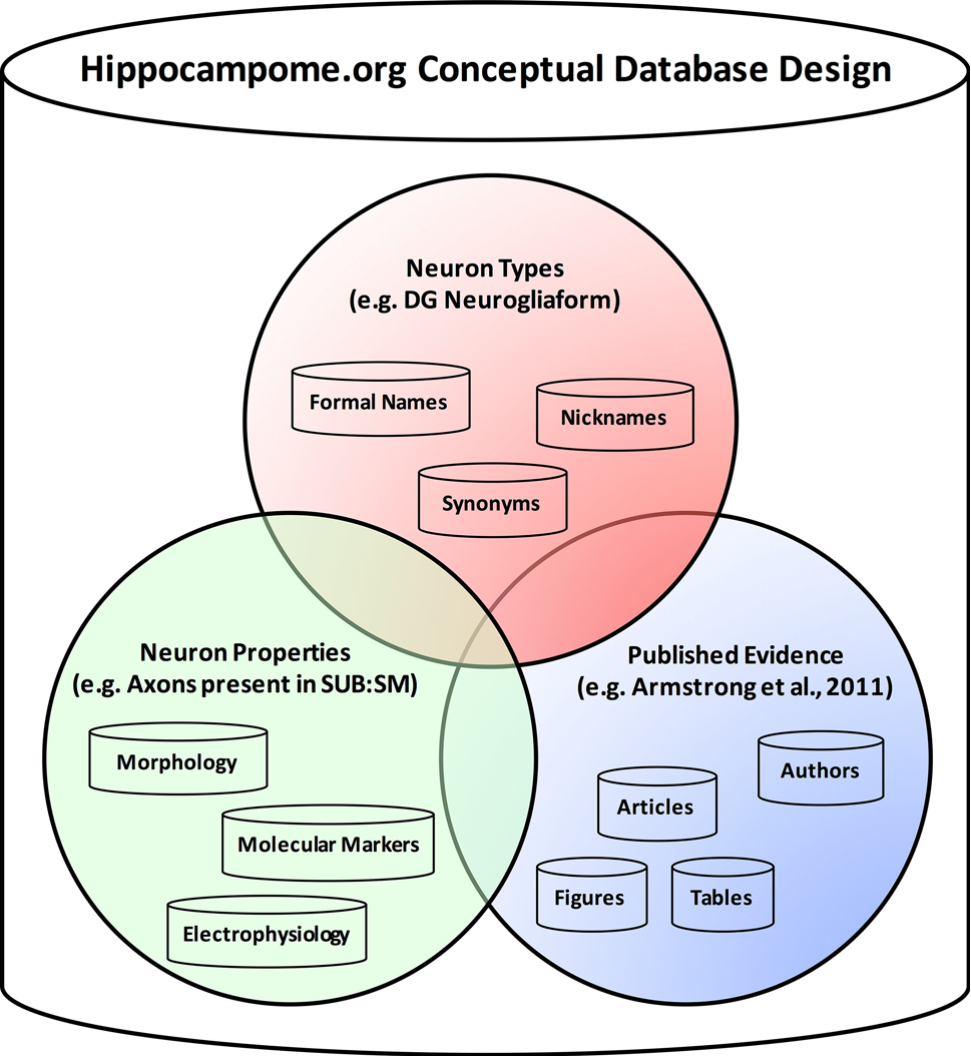
Hippocampome.org conceptual design. The database groups information into three general categories: neuron types, neuron properties, and published evidence. Links between data and relations are captured in separate relation tables to both increase flexibility and reduce complexity, therefore facilitating maintainability

We designed an open-source online system enabling machine-readable information accessibility. Knowledge about each Hippocampome.org neuron type, including the names, synonyms, properties, and evidence, is stored in a relational database sourcing a user-friendly web-accessible interface. Figure 3 depicts the conceptual organization of the database based on three general categories: neuron types, neuron properties, and published evidence. Links between data and relations are captured in separate relation tables, to both increase flexibility and reduce complexity, thereby facilitating continuous development and long-term maintainability.

Converting information published for human consumption into machine-readable form dictates system level decisions to minimize the energy cost of processing. We chose a three-step workflow. The first step is for researchers (doctoral students, postdocs, and faculty) to identify and study relevant articles, gleaning salient information and encoding it into spreadsheets. The second step involves python code to ingest these spreadsheets into data tables, populating along the way relation tables. The third step consists of rendering the resulting structured data in web pages dynamically leveraging the database. Performing the most time consuming tasks up front (steps one and two) allows for fast web-based lookup access by the end-user community. The data/relation table design adds a layer of complexity to the database, but simplifies the resultant query implementation complexity, considerably speeding up real-time interactive retrieval.

### Neuron term machine-readable definition identifier

**Fig. 4:**
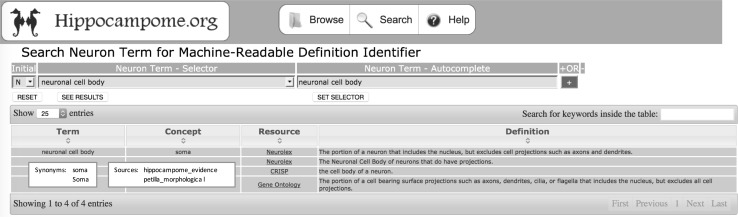
Neuron term machine-readable definition identifier: an online portal for conceptual mapping of neuronal properties fully integrated in Hippocampome.org

In order to facilitate the collation of machine-readable definitions of relevant terms, we designed and implemented a novel functionality of Hippocampome.org for online assistance in disambiguating neuron property nomenclature (Fig. 4). This new resource (Hippocampome.org) integrates key neuron term descriptors into a curated catalog of web-accessible human- and machine-readable definitions. Users can browse, search, and filter terms from drop-down menus augmented with autocomplete-as-you-type function. After selecting one or more terms, the portal returns the mapped concept with mouse/cursor-layover display of all available synonyms and the context in which they appear, along with a list of available definitions and direct hyperlinks to the corresponding source providers. Users can also search for specific keywords of interest within the definitions. Furthermore, when browsing Hippocampome.org and all cited evidence within, terms with available definitions are now highlighted: users can display a definition pop-up with mouse/cursor-layover or directly click on the term for linking out to the corresponding entry from the providing resource.

**Fig. 5:**
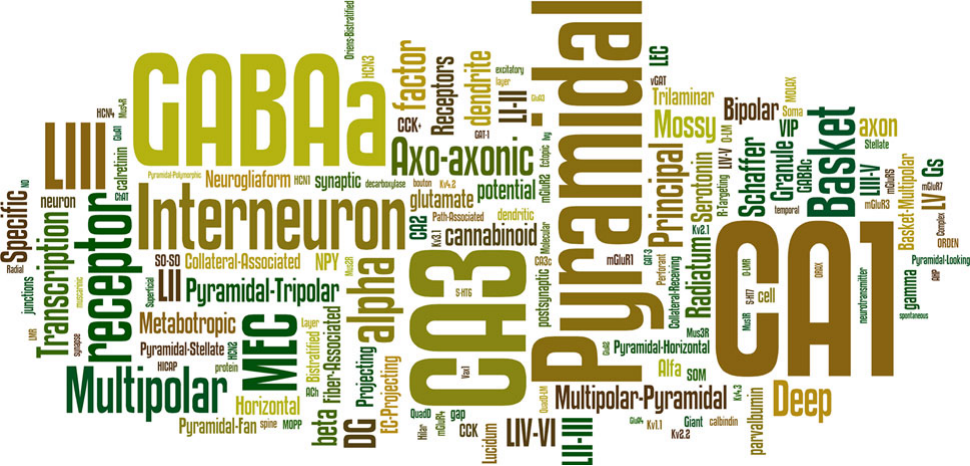
Word cloud of representative Hippocampome.org terms

The first challenge in deploying this novel functionality was to identify the set of terms requiring machine-readable definitions. This research leveraged two primary sources of terms: Petilla [4] and the article excerpts cited as evidence in Hippocampome.org [11]. The Neuron Registry [7] constituted a third minor source of terms. The Petilla terminology consists of a finite list of (~232) published terms. Hippocampome.org, in contrast, contributes a less neatly bounded set of terms exceeding 10 K discrete tokens (as estimated by the wordle.net utility, Fig. 5). To parse these tokens into a manageable set, we filtered the Hippocampome.org terms at each extreme of the occurrence count spectrum. This preprocessing step eliminated words with very large (>1000) occurrence counts, including uninformative strings such as “a,” “the,” and “of,” as well as words with very small (<100) occurrence counts, representing rare and typically uninteresting terms like “outside-out” and “sheetlike.” Lastly we hand-curated the remaining set of approximately 700 terms to remove non-scientifically relevant words yielding a final corpus of 490 evidence-derived terms. An additional 782 terms corresponded to neuron names, anatomical regions, biomarkers, and electrophysiological parameters stored in Hippocampome.org. In all, due to minor overlaps among the above lists, this collation accounted for 1478 distinct terms.

To find machine-readable definitions we devised a preferred portal/repository approach. For general neurobiological terms, we first searched Neurolex.org, MeSH browser (nlm.nih.gov/mesh), the Bioportal services from the National Center for Biomedical Ontology [42], and the US Public Health Service CRISP database [43]. The terms from Hippocampome.org evidence primarily refer to the rodent hippocampus, thus it is essential that the extracted definition be relevant to these target domains. Since the same word can have different meanings, most definitions retrieved by the initial automated search were largely out of context, requiring a slow step of manual curation. We preferentially assigned evidence terms from Hippocampome.org definitions and links most relevant to the rodent hippocampal formation. Similarly, we linked the Petilla terms to definitions in the context of GABAergic interneurons of the cerebral cortex.

For protein definitions, we harnessed the Ontology Look-up Service [44] of the Gene Ontology Consortium [45] as the sole reference given the depth and breadth of coverage for this type of molecular data. Because the molecular terms are generally regular and systematically databased, we successfully automated API-based pulling from established sources (e.g., the National Center for Biotechnology Information). For term not found in these primary resources, we reverted to Google searches, prioritizing definitions from scholarly or institutional sources such as the Allen Brain Atlas [46], Scholarpedia.org, and the US National Institute of Standards and Technology (nist.gov). For residual blanks, we resorted to dictionaries like Merriam-Webster or Wikipedia.

The last step of manual curation involved concept mapping to group together distinct terms linking to textually different but logically analogous definitions. For example, “action potential” and “spike” are synonyms for which multiple machine-readable definitions exist. This mapping yielded 810 distinct concepts from the 1478 unique terms, with a total of 924 unique definitions from 1378 distinct resource links. Table 1 summarizes the neuron term counts, including number per category (i.e., morphological, molecular, and electrophysiological) and unique instances. Table 2 organizes this information by resources providing the machine-readable external links to the term definitions.

**Table 1:** Neuron term summary and examples

Category	Term source	Summary				Examples			
		Terms	Concepts	External links	Definitions	Terms	Concepts	External links	Definitions
Neuron type	Hippocampome.org	641	122	1	122	CA1 ivy interneuron	CA1 Ivy	http://hippocampome.org/formal-typedefs	Inhibitory neuron with axons in [CA1:SR CA1:SP CA1:SO], dendrites in [CA1:SR CA1:SP CA1:SO], and soma in [CA1:SR or CA1:SP or CA1:SO]; PV- SOM- nNOS+
Morphology	Petilla	38	41	75	64	Soma - Shape -Triangular	Pyramidal	http://neurolex.org/wiki/Category:Pyramidal	Generally triangular-shaped soma with a dendrite emerging from the apical pole and basilar dendrites emerging from two or more basal poles
	Hippocampome.org	34	34	32	33	CA1:SP	CA1:SP	http://atlas.brain-map.org/#atlas=1&plate=100960224&structure=399&x=4594.285714285715&y=3595.428641183036&zoom=-3&resolution=11.97&z=5	Field CA1, pyramidal layer
Molecular marker	Petilla	175	176	752	183	Transcription factor - Nkx2.1	Transcription factor - Nkx2.1	http://www.ncbi.nlm.nih.gov/gene/25628	Transcription factor that binds and activates the promoter of thyroid specific genes such as thyroglobulin, thyroperoxidase, and thyrotropin receptor; may play a role in organogenesis and/or reproductive maturation and development [RGD, Feb 2006]
	Hippocampome.org	97	95	95	95	PV	Parvalbumin	http://nakama.berkeleybop.org/amigo/gene_product/MGI:MGI:97821	parvalbumin; Symbol:Pvalb; Type:protein; Taxon:Mus musculus; Synonyms: Parv, PV, Pva
Electrophysiology	Petilla	19	20	36	28	Spike amplitude	APampl	http://bioportal.bioontology.org/ontologies/42295?p=terms&conceptid=X77KG	Nerve action potential amplitude
	Hippocampome.org	10	10	8	14	APampl	APampl	http://neurolex.org/wiki/Category:Spike_Amplitude	Average amplitude of the first AP (measured from AP threshold to AP peak)
Evidence & notes	Hippocampome.org	490	429	434	436	Gap junction	Electrical synapse	http://www.nlm.nih.gov/cgi/mesh/2011/MB_cgi?mode=&term=Electrical+Synapses&field=entry	Specialized junctions between neurons which connect the cytoplasm of one neuron to another allowing direct passage of an ion current
	Sum	1504	1049	1434	1097				
	Distinct	1478	810	1378	924

**Table 2:** Term resource summary

Rank	Resource	Terms	Concepts	Definitions
1	BIOPORTAL	53	53	32
	CRISP	33	33	30
	Gene ontology	119	100	105
	Hippocampome.org	649	130	130
	MeSH	176	168	111
	NCBI gene	112	111	6
	NCBI protein	103	102	7
	Neurolex	429	354	311
	Protein ontology	121	120	6
2	Allen Brain Atlas	35	35	33
	ChEBI	1	1	1
	MBF bioscience	1	1	1
	Medical College of Wisconsin	1	1	1
	Merriam-Webster medical	69	68	68
	NeuroElectro	1	1	1
	NIST InChI trust	1	1	1
	Scholarpedia	6	6	6
	UTHealth neuroscience	1	1	1
	Wolfram mathworld	4	3	3
3	Cambridge dictionaries	1	1	1
	Macmillan dictionary	1	1	1
	Merriam-Webster dictionary	39	38	38
	Oxford dictionaries	2	2	2
	TheFree dictionary	22	20	20
	Wikipedia	16	16	14
	Wiktionary	5	5	5
	Sum	2001	1372	935
	Distinct	1478	810	924

### Neuron type naming

The classification schema introduced by Hippocampome.org [11] defines neuron types based on their properties, starting from morphological patterns and with the added specification of molecular and electrophysiological features. For example, Hippocampome.org defines dentate gyrus granule cells as excitatory neurons with axons in the hilus, CA3 lucidum/pyramidale, and CA2 pyramidale, dendrites in the inner and outer molecular layer, and soma in the granular layer. These definitions are now available as an explicit list (hippocampome.org/neuron-types) and linked from the term definition portal described above.

It is difficult to quantify how many unique neuron types have been defined to date in the hippocampal formation due to ambiguity and overlap of descriptors across research labs. We constrain the number of Hippocampome.org [11] neuron types (e.g., 122 in the initial release) by limiting the primary characterization properties to axonal/dendritic patterns and excitatory/inhibitory neurotransmitters.

**Fig. 6:**
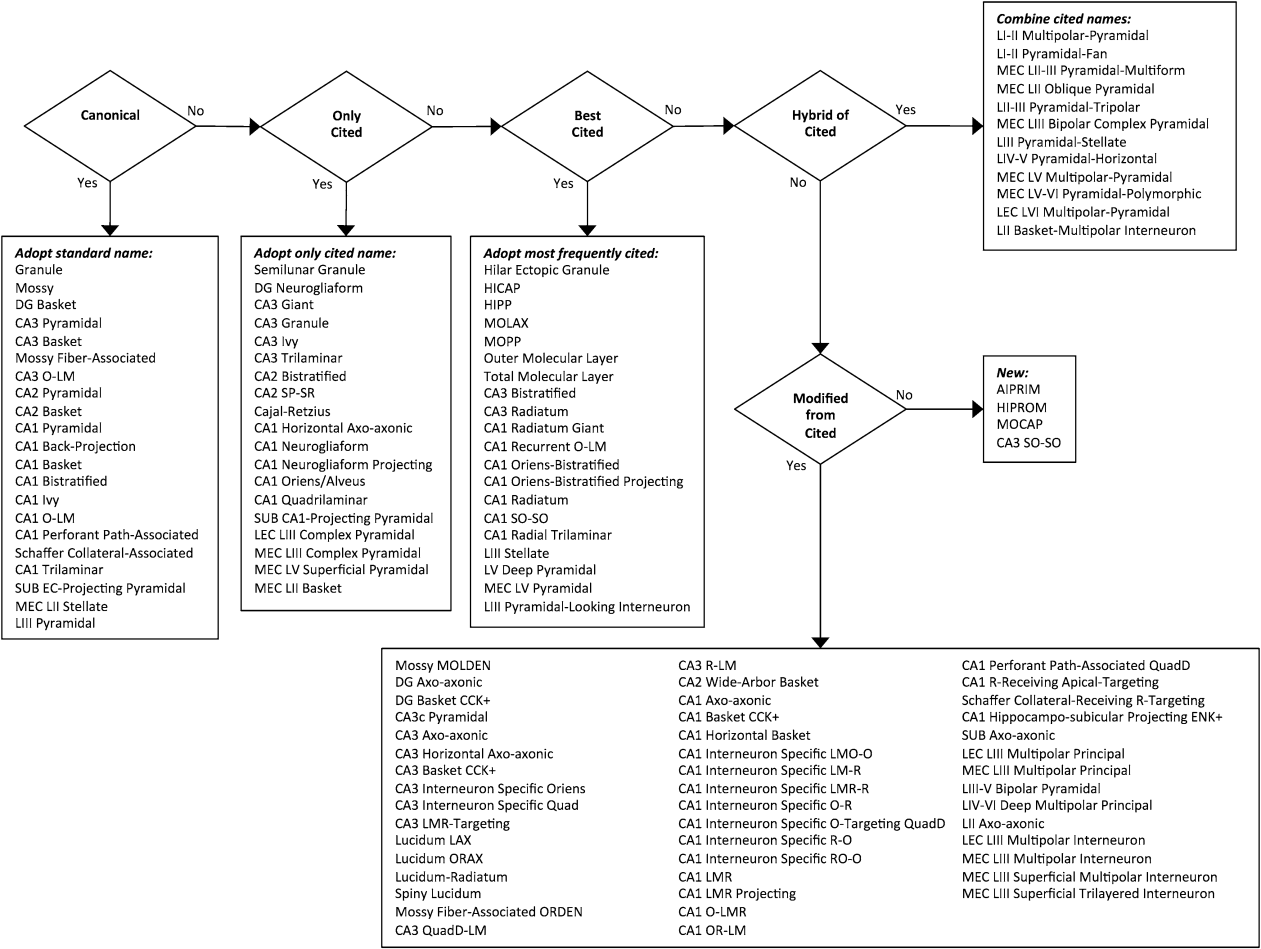
Decision logic for assigning common names to Hippocampome.org neuron types

Furthermore, Hippocampome.org neuron types are assigned both a formal name and a unique number identifier (e.g., DG (e) 2201p-CA3_00110 Granule; type 1000). The formal name contains several components (hippocampome.org/formal-name): (a) the abbreviation of the subregion where the soma is located, (b) a symbol specifying the putative major neurotransmitter (i.e., “e” for glutamatergic, excitatory neurons or “i” for GABAergic, inhibitory neurons), and (c) a numeric encoding for the presence or absence of neurites within the subregion of soma location. In neuron types whose axons extend outside of their home subregion, the numerical encoding continues with a “p” (for projecting) followed by codes analogous to (a) and (c) to specify the subregions receiving the projection. Finally, the formal name ends with a unique, human-friendly label that attempts to maximize usability and understanding of neuron types within the research community. Figure 6 illustrates the selection process for determining this “common name.”

In the most clear-cut cases, a single name dominates the literature as universally recognized and understood. In such “canonical” cases, we adopt these standard names, as in Granule, Mossy, CA3 basket, and CA1 pyramidal cells. In other situations, a neuron type may not be as broadly known, but is only cited in a single way. In these cases, we straightforwardly adopt the single cited name, such as in Semilunar Granule, CA3 Giant, and CA3 Granule cells. The remaining cases represent the confusing scenarios in which the literature describes the same neuron types with multiple names and different neuron types with the same name.

If one name or acronym is clearly dominant, with more frequent citations than all other names, we adopt it as the common name, as in the cases of HIPP, MOPP, HICAP, and MOLAX interneurons. Other neuron types, however, have multiple, approximately equally cited names, especially in the less-studied entorhinal cortex. In these cases, to avoid playing favorites, we hybridize the cited names, as is LI-II Multipolar-Pyramidal, LI-II Pyramidal-Fan, and MEC LII-III Pyramidal-Multiform. Lastly, there are neuron types for which all cited names entail potential confusion with similar or identical names already assigned to other neuron types based upon the rules above. In these scenarios, we are forced to either modify a cited name in order to differentiate it (e.g., Mossy MOLDEN, DG Basket CCK+, and CA3c Pyramidal) or to create a new name altogether (e.g., AIPRIM, HIPROM, MOCAP, CA3 SO–SO). We try to use this final clause sparingly (only 4 names out of 122 in Hippocampome.org are entirely new), but minor modifications of pre-existing names are often unavoidable (46 out of 122).

## Discussion

The basis of communication is language. Unfortunately, the language of neuroscience is lacking a common terminology with respect to neuron types and their associated discriminating properties. Paraphrasing Shakespeare: “What’s in a name? That which we call a [neuron] by any other name would [fire] as [frequently].” By first establishing neuron types based on their necessary and sufficient common characteristics, and then methodically applying a naming protocol, it is possible to establish a basis for systematic neuron naming. This work differs from prior efforts in the level of comprehensiveness. There have not been any all-inclusive compilations of neuron types within the entire rodent hippocampal formation based on peer-reviewed published literature for the past two decades [3]. Scientific laboratories most often work independently, and researchers performing experiments typically name neurons for their convenience. Hippocampome.org dynamically integrates these data across all known experimental evidence.

We have striven to find human-friendly names that are recognizable to, at the least, those who are familiar with hippocampal neurons. In many cases, however, these names have minimal informational content to those unfamiliar with the type. The part of the formal name that is most informative is the numeric encoding of the neurite pattern (detailed description: hippocampome.org/find-term). Knowledge of the pattern of dendrites and axons confers information about potential connectivity of the neuron type within the circuit. Therefore, incorporation of this pattern into the name allows instantaneous envisioning of the location of the neurites and by extension the connectivity of the type. In addition, this numeric encoding is unique for most neuron types with only subtypes discriminated by their primary neurotransmitter, post-synaptic target specificity, or molecular marker and/or electrophysiology profiles having the same pattern. In these cases, the human-friendly part of the name provides uniqueness (e.g., “CA1 2232 Basket” and “CA1 2232 Basket CCK+”). This method of naming neurons results in extremely informative, concise names without necessitating the memorization of many acronyms. Furthermore, it is applicable to any brain region that is divisible into parcels.

Going beyond Hippocampome.org, the same approach to defining neuron types can be extended outside the hippocampal formation. For example, CA1 neurons that project to other brain regions such as the lateral septum, medial septum, and/or hypothalamus can be characterized by extending the axonal/dendritic patterns to encompass those regions.

Nomenclature confusion could be mitigated with increased awareness of the neurons, molecules, and properties and how they fit in the historical context. This is a lot to ask of researchers, but resources like Hippocampome.org provide significant assistance. Hippocampome.org demonstrates that the necessary and sufficient discriminating property of neurite patterning is a workable and advantageous foundation upon which to build a neuron type library. Enhancing such a library with a terms definition portal further reduces terminology confusion. Coupled, these resources begin clarifying the muddied state of the literature and re-illuminating the path to neuroscience progress.

## References

[1] Kushchayev SV, Moskalenko VF, Wiener PC, Tsymbaliuk VI, Cherkasov VG, Dzyavulska IV, Kovalchuk OI, Sonntag VK, Spetzler RF, Preul MC (2012). The discovery of the pyramidal neurons: Vladimir Betz and a new era of neuroscience. Brain.

[2] Elston GN (2003). Cortex, cognition and the cell: new insights into the pyramidal neuron and prefrontal function. Cereb Cortex.

[3] Freund TF, Buzsáki G (1996). Interneurons of the hippocampus. Hippocampus.

[4] Ascoli GA, Alonso-Nanclares L, Anderson SA, Barrionuevo G, Benavides-Piccione R, Burkhalter A, Buzsáki G, Cauli B, Defelipe J, Fairén A, Feldmeyer D, Fishell G, Fregnac Y, Freund TF, Gardner D, Gardner EP, Goldberg JH, Helmstaedter M, Hestrin S, Karube F, Kisvárday ZF, Lambolez B, Lewis DA, Marin O, Markram H, Muñoz A, Packer A, Petersen CC, Rockland KS, Rossier J, Rudy B, Somogyi P, Staiger JF, Tamas G, Thomson AM, Toledo-Rodriguez M, Wang Y, West DC, Yuste R (2008). Petilla terminology: nomenclature of features of GABAergic interneurons of the cerebral cortex. Nat Rev Neurosci.

[5] Bowden DM, Song E, Kosheleva J, Dubach MF (2012). NeuroNames: an ontology for the BrainInfo portal to neuroscience on the web. Neuroinformatics.

[6] Bug WJ, Ascoli GA, Grethe JS, Gupta A, Fennema-Notestine C, Laird AR, Larson SD, Rubin D, Shepherd GM, Turner JA, Martone ME (2008). The NIFSTD and BIRNLex vocabularies: building comprehensive ontologies for neuroscience. Neuroinformatics.

[7] Hamilton DJ, Shepherd GM, Martone ME, Ascoli GA (2012). An ontological approach to describing neurons and their relationships. Front Neuroinform.

[8] Armañanzas R, Ascoli GA (2015). Towards the automatic classification of neurons. Trends Neurosci.

[9] Battaglia D, Karagiannis A, Gallopin T, Gutch HW, Cauli B (2013). Beyond the frontiers of neuronal types. Front Neural Circuits.

[10] DeFelipe J, López-Cruz PL, Benavides-Piccione R, Bielza C, Larrañaga P, Anderson S, Burkhalter A, Cauli B, Fairén A, Feldmeyer D, Fishell G, Fitzpatrick D, Freund TF, González-Burgos G, Hestrin S, Hill S, Hof PR, Huang J, Jones EG, Kawaguchi Y, Kisvárday Z, Kubota Y, Lewis DA, Marín O, Markram H, McBain CJ, Meyer HS, Monyer H, Nelson SB, Rockland K, Rossier J, Rubenstein JL, Rudy B, Scanziani M, Shepherd GM, Sherwood CC, Staiger JF, Tamás G, Thomson A, Wang Y, Yuste R, Ascoli GA (2013). New insights into the classification and nomenclature of cortical GABAergic interneurons. Nat Rev Neurosci.

[11] Wheeler DW, White CM, Rees CL, Komendantov AO, Hamilton DJ, Ascoli GA (2015). Hippocampome.org: a knowledge base of neuron types in the rodent hippocampus. Elife.

[12] Kumar SS, Buckmaster PS (2006). Hyperexcitability, interneurons, and loss of GABAergic synapses in entorhinal cortex in a model of temporal lobe epilepsy. J Neurosci.

[13] Lingenhöhl K, Finch DM (1991). Morphological characterization of rat entorhinal neurons in vivo: soma-dendritic structure and axonal domains. Exp Brain Res.

[14] Canto CB, Witter MP (2012). Cellular properties of principal neurons in the rat entorhinal cortex. II. The medial entorhinal cortex. Hippocampus.

[15] Buhl EH, Halasy K, Somogyi P (1994). Diverse sources of hippocampal unitary inhibitory postsynaptic potentials and the number of synaptic release sites. Nature.

[16] Daw MI, Tricoire L, Erdelyi F, Szabo G, McBain CJ (2009). Asynchronous transmitter release from cholecystokinin-containing inhibitory interneurons is widespread and target-cell independent. J Neurosci.

[17] Sik A, Penttonen M, Ylinen A, Buzsáki G (1995). Hippocampal CA1 interneurons: an in vivo intracellular labeling study. J Neurosci.

[18] Vida I, Halasy K, Szinyei C, Somogyi P, Buhl EH (1998). Unitary IPSPs evoked by interneurons at the stratum radiatum-stratum lacunosum-moleculare border in the CA1 area of the rat hippocampus in vitro. J Physiol.

[19] Klausberger T, Marton LF, O’Neill J, Huck JH, Dalezios Y, Fuentealba P, Suen WY, Papp E, Kaneko T, Watanabe M, Csicsvari J, Somogyi P (2005). Complementary roles of cholecystokinin- and parvalbumin-expressing GABAergic neurons in hippocampal network oscillations. J Neurosci.

[20] Hájos N, Papp EC, Acsády L, Levey AI, Freund TF (1998). Distinct interneuron types express m2 muscarinic receptor immunoreactivity on their dendrites or axon terminals in the hippocampus. Neuroscience.

[21] Zemankovics R, Káli S, Paulsen O, Freund TF, Hájos N (2010). Differences in subthreshold resonance of hippocampal pyramidal cells and interneurons: the role of h-current and passive membrane characteristics. J Physiol.

[22] Svoboda KR, Adams CE, Lupica CR (1999). Opioid receptor subtype expression defines morphologically distinct classes of hippocampal interneurons. J Neurosci.

[23] Ali AB, Thomson AM (1998). Facilitating pyramid to horizontal oriens-alveus interneurone inputs: dual intracellular recordings in slices of rat hippocampus. J Physiol.

[24] Losonczy A, Zhang L, Shigemoto R, Somogyi P, Nusser Z (2002). Cell type dependence and variability in the short-term plasticity of EPSCs in identified mouse hippocampal interneurones. J Physiol.

[25] Hájos N, Mody I (1997). Synaptic communication among hippocampal interneurons: properties of spontaneous IPSCs in morphologically identified cells. J Neurosci.

[26] Amaral D, Lavenex P, Andersen P, Morris R, Amaral D, Bliss T, OKeefe J (2007). Hippocampal neuroanatomy. The hippocampus book.

[27] Lasztóczi B, Tukker JJ, Somogyi P, Klausberger T (2011). Terminal field and firing selectivity of cholecystokinin-expressing interneurons in the hippocampal CA3 area. J Neurosci.

[28] Gulyás AI, Miles R, Hájos N, Freund TF (1993). Precision and variability in postsynaptic target selection of inhibitory cells in the hippocampal CA3 region. Eur J Neurosci.

[29] Ascoli GA, Brown KM, Calixto E, Card JP, Galván EJ, Perez-Rosello T, Barrionuevo G (2009). Quantitative morphometry of electrophysiologically identified CA3b interneurons reveals robust local geometry and distinct cell classes. J Comp Neurol.

[30] Mott DD, Turner DA, Okazaki MM, Lewis DV (1997). Interneurons of the dentate-hilus border of the rat dentate gyrus: morphological and electrophysiological heterogeneity. J Neurosci.

[31] Lübke J, Frotscher M, Spruston N (1998). Specialized electrophysiological properties of anatomically identified neurons in the hilar region of the rat fascia dentata. J Neurophysiol.

[32] Szabadics J, Soltesz I (2009). Functional specificity of mossy fiber innervation of GABAergic cells in the hippocampus. J Neurosci.

[33] Hamam BN, Amaral DG, Alonso AA (2002). Morphological and electrophysiological characteristics of layer V neurons of the rat lateral entorhinal cortex. J Comp Neurol.

[34] Kiss J, Csaba Z, Csáki A, Halász B (2013). Demonstration of estrogen receptor α protein in glutamatergic (vesicular glutamate transporter 2 immunoreactive) neurons of the female rat hypothalamus and amygdala using double-label immunocytochemistry. Exp Brain Res.

[35] Lei W, Deng Y, Liu B, Mu S, Guley NM, Wong T, Reiner A (2013). Confocal laser scanning microscopy and ultrastructural study of VGLUT2 thalamic input to striatal projection neurons in rats. J Comp Neurol.

[36] Yokoyama T, Nakamuta N, Kusakabe T, Yamamoto Y (2014). Vesicular glutamate transporter 2-immunoreactive afferent nerve terminals in the carotid body of the rat. Cell Tissue Res.

[37] Favier M, Carcenac C, Drui G, Boulet S, El Mestikawy S, Savasta M (2013). High-frequency stimulation of the subthalamic nucleus modifies the expression of vesicular glutamate transporters in basal ganglia in a rat model of Parkinson’s disease. BMC Neurosci.

[38] Dingledine R, Borges K, Bowie D, Traynelis SF (1999). The glutamate receptor ion channels. Pharmacol Rev.

[39] Traynelis SF, Wollmuth LP, McBain CJ, Menniti FS, Vance KM, Ogden KK, Hansen KB, Yuan H, Myers SJ, Dingledine R (2010). Glutamate receptor ion channels: structure, regulation, and function. Pharmacol Rev.

[40] Yepes AJJ, Plaza L, Carrillo-de-Albornoz J, Mork JG, Aronson AR (2015). Feature engineering for MEDLINE citation categorization with MeSH. BMC Bioinform.

[41] Larson SD, Martone ME (2013). NeuroLex.org: an online framework for neuroscience knowledge. Front Neuroinform.

[42] Whetzel PL, Noy NF, Shah NH, Alexander PR, Nyulas C, Tudorache T, Musen MA (2011). BioPortal: enhanced functionality via new Web services from the National Center for Biomedical Ontology to access and use ontologies in software applications. Nucleic Acids Res.

[43] Collins KA (1989). The CRISP system: an untapped resource for biomedical research project information. Bull Med Libr Assoc.

[44] Côté R, Reisinger F, Martens L, Barsnes H, Vizcaino JA, Hermjakob H (2010). The Ontology Lookup Service: bigger and better. Nucleic Acids Res.

[45] Gene Ontology Consortium (2015). Gene Ontology Consortium going forward. Nucleic Acids Res.

[46] Somogyi P, Klausberger T (2005). Defined types of cortical interneurone structure space and spike timing in the hippocampus. J Physiol.

[47] Shen EH, Overly CC, Jones AR (2012). The Allen Human Brain Atlas: comprehensive gene expression mapping of the human brain. Trends Neurosci.

